# Tamoxifen treatment causes early hepatic insulin resistance

**DOI:** 10.1007/s00592-019-01468-6

**Published:** 2020-01-06

**Authors:** Nora Klöting, Matthias Kern, Michele Moruzzi, Michael Stumvoll, Matthias Blüher

**Affiliations:** 1 Helmholtz Institute for Metabolic, Obesity and Vascular Research (HI-MAG) of the Helmholtz Zentrum München, University of Leipzig and University Hospital Leipzig, Liebigstr. 20, 04103 Leipzig, Germany; 2 IFB AdiposityDiseases, University of Leipzig, Leipzig, Germany; 3 German Diabetes Center, University of Leipzig, Leipzig, Germany; 4 Department of Medicine, University of Leipzig, Leipzig, Germany

## Introduction

Tamoxifen is widely used as an effective adjuvant treatment and prevention of estrogen receptor-positive breast cancer [1]. Tamoxifen decreases food intake, body weight and (at least in the short-term) fat mass in rodents. Despite lowering body weight in obese women, tamoxifen may increase the incidence of diabetes [2]. This potential side effect has been attributed to decreased *β*-cell survival and proliferation, but also hepatic steatosis in rodents [3]. We and others have recently demonstrated that tamoxifen affects glucose, lipid metabolism, transient body composition changes which may originate from tamoxifen-induced adipose tissue necrosis followed by de novo adipocyte recruitment and fat redistribution including fat accumulation in the liver in the longer term [3, 4]. Indeed, the development of fatty liver was observed as early as 3 months after initiation of tamoxifen treatment in women with breast cancer [5]. However, the precise mechanisms of potential diabetogenic effects of tamoxifen are still not entirely clear. Therefore, we aimed to systematically elucidate the development of tamoxifen-related hyperglycemia using the hyperinsulinemic–euglycemic clamps.

## Methods

### Animal experiments

All animal studies were approved by the local authorities of the state of Saxony, Germany as recommended by the responsible local animal ethics review board (Landesdirektion Leipzig, TVV21/12, T03/13 Germany). Twelve-week-old female C57BL/6NTac mice (*N* = 8) were housed in on a 12-h light/dark cycle with water and food (standard chow, Sniff Spezialdiäten, Soest, Germany) ad libitum. Animals were bred and kept in the Animal Laboratories at the University of Leipzig. Tamoxifen (Tam, Sigma‐Aldrich, Taufkirchen, Germany, #T5648) was solved in triglyceride oil (Fagron, Barsbüttel, Germany, #700,282) and was injected intraperitoneally 1 mg per day in five consecutive days. Controls were injected with solvent only. After 24 days of Tam treatment, catheters were implanted in the left jugular vein. Hyperinsulinemic–euglycemic clamps were performed in four female mice of each experimental group as described [3]. Briefly, the hyperinsulinemic–euglycemic clamp was conducted with a continuous infusion of human insulin at a rate of 20 mU/kg/min to lower plasma glucose levels within the physiological range (~ 5 mmol/l). Blood glucose concentrations were maintained by adjusting infusion rates of a 20% glucose solution. Steady state was defined as constant glucose levels for 20 min at a stable glucose infusion rate (GIR) which was achieved within 120–240 min. Steady state was maintained for 45 min, and blood samples (10 μL) were taken at baseline, every 5 min, and every 10 min during steady state. All infusions were applied using micro-dialysis pumps (TSE Systems, Chesterfield, MO, USA). Hepatic glucose production (HPG, mg × kg^−1^ × min^−1^) was calculated as the difference between the rate of glucose appearance and glucose infusion rate. Immediately after the clamp, mice were killed by cervical dislocation and liver, subcutaneous (SC), epigonadal (EPI) and interscapular brown fat were extracted and weighed.

Before performing clamp studies, blood glucose values were determined from whole venous blood samples using an automated analyzer (COBAS 8000, Roche Diagnostics, Mannheim, Germany). Serum insulin concentrations were measured by ELISA using mouse standards according to the manufacturer’s guidelines (Insulin ELISA; CrystalChem. Inc, Downers Grove, IL). In a second subset of animals (*N* = 6 per treatment group), serum concentrations of triglycerides (TG), free fatty acids (FFA), total cholesterol (Chol) and aspartate aminotransferase (AST) were analyzed using the COBAS 8000 modular analyzer (Roche Diagnostics, Mannheim, Germany).

Hepatic 4 µm sections were stained with hematoxylin and eosin (H&E) for routine examination for detection of liver tissue-infiltrating leukocytes.

## Statistical analyses

Prism 6.0 software (GraphPad Software) was used for statistical analyses. Data are given as mean ± SE. Data sets were analyzed for statistical significance using a two-tailed unpaired Student *t* test or Mann–Whitney *U* test. *P* values < 0.05 were considered significant.

## Results

**Fig. 1:**
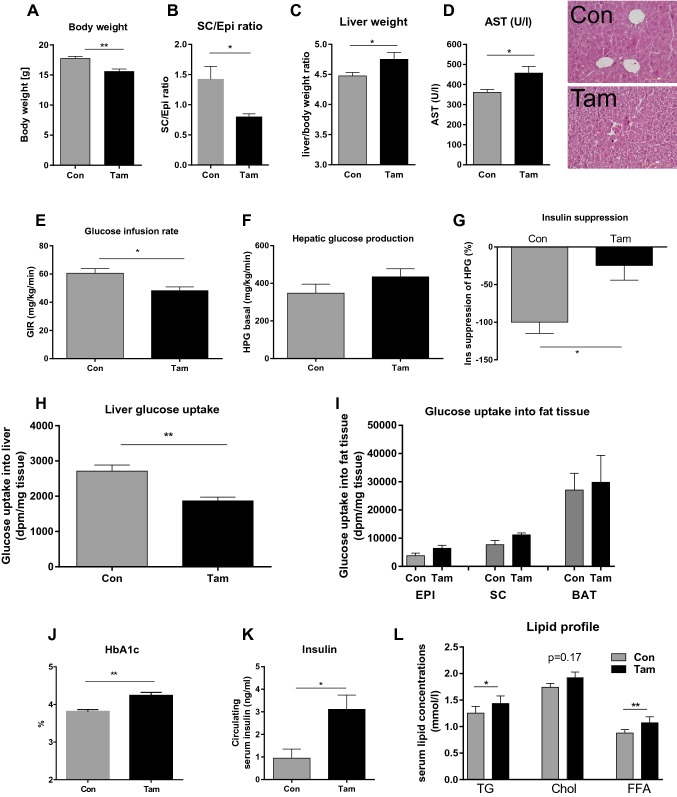
Tamoxifen treatment effects on body composition, liver and glucose metabolism in female C57BL/6 N mice 12-weeks old. **a** Body weight after 24 days of 5 day treatment of 1 mg/kg tamoxifen (Tam) or oil controls (con) **b** subcutaneous/epigonadal fat ratio [3]; **c** relative liver weight; **d** aspartate aminotransferase (AST) concentrations and representative images of hepatic tissue of controls (Con) and tamoxifen treated (Tam) (original magnification × 200) showing hepatocellular injury with ballooning cells of Tam-treated mice. 4 µm sections were stained with hematoxylin and eosin (H&E) for routine examination for detection of liver tissue-infiltrating leukocytes. **e** Glucose infusion rate (GIR, mg/kg/min) during a hyperinsulinemic–euglycemic clamp was decreased in tamoxifen (Tam)-treated mice (*N* = 4) compared with control (oil-treated) mice (N = 4) 24 days after Tam treatment. Results are expressed as means ± SE. **f** Basal hepatic glucose production (HPG) during the clamp in Tam mice is comparable to controls (*N* = 4). Hepatic glucose production (mg × kg^1^  × min^1^) was calculated as the difference between the rate of glucose appearance and glucose infusion rate (GIR). Results are expressed as means ± SE from four animals per treatment group **g** percentage suppression of hepatic glucose production by insulin during the clamp in Tam (*N* = 4) and control mice (*N* = 4) indicating a decreased ability of insulin to suppress HPG production. Results are expressed as means ± SE from four animals per group. **h** Significantly reduced glucose uptake into liver in Tam-treated mice compared to controls and **i** glucose uptake into fat tissue (EPI, epigonadal; SC subcutaneous; BAT, brown adipose tissue) **j** HbA1c levels in percent **k** circulating serum insulin levels, **l** serum triglyceride (TG), total cholesterol (Chol) and free fatty acids (FFA) concentrations. Results are expressed as means ± SE from at least four animals per treatment group. The different degrees of significance are indicated as follows: *P < 0.05; **P < 0.01

We performed hyperinsulinemic–euglycemic clamp studies in healthy lean mice in the weight regain phase following the previously reported tamoxifen-induced weight loss (14 days after the first tamoxifen treatment dose, Fig. 1a). The weight regain is associated with increased ectopic fat deposition including the development of fatty liver and increased relative liver weight (Fig. 1b, c). In addition, AST serum concentration is significantly higher in tamoxifen-treated mice (Fig. 1d). Tamoxifen-treated mice are characterized by impaired insulin sensitivity at the whole body (Fig. 1e) and liver level (Fig. 1f–h). Glucose infusion rate and the ability of insulin to reduce hepatic glucose production were significantly lower in tamoxifen treated compared with control mice (Fig. 1g). Liver glucose uptake was significantly lower in tamoxifen-treated mice (Fig. 1h), whereas adipose tissue was not affected (Fig. 1i). HbA1c (Fig. 1j), fasted insulin (Fig. 1k), triglyceride and free fatty acid serum concentrations (Fig. 1l) were significantly higher in tamoxifen-treated mice compared to controls (Fig. 1).

## Discussion

Here, we show that our previously reported tamoxifen treatment model elucidates the sequence of pathomechanisms underlying the diabetogenic side effect of tamoxifen [4]. In mice, tamoxifen acutely caused a ~ 75% loss of body fat which was followed by a weight regain phase starting within the first weeks [4]. During this period, we performed hyperinsulinemic–euglycemic clamp studies in healthy lean mice. Weight regain lead to increased ectopic fat deposition including the development of fatty liver and its associated disturbances in liver function tests (AST) and circulating lipid profile (Fig. l), suggesting that the capacity of healthy adipose tissue storage is exceeded by the rapid regain. As a symptom of impaired adipose tissue expandability, glucose uptake into adipose tissue was not altered during weight regain (Fig. 1i). Importantly, fat redistribution was associated with impaired insulin sensitivity both at the systemic and hepatic level (Fig. 1e–h). A combination of liver insulin resistance with reduced liver glucose uptake may contribute to the subsequent development of hyperglycemia (Fig. 1j) and hyperinsulinemia (Fig. 1k) in mice recovering from the acute body fat loss upon tamoxifen treatment. However, a causal relationship between liver fat accumulation and the development of diabetes cannot be established based on our experiments. Taken together, these data support a concept that the mechanism for the tamoxifen-associated development of diabetes maybe mediated through an early and rapid increase in liver fat. We propose that this process starts with an inability of acutely depleted adipose tissue to compensate for the rapid weight regain leading to ectopic fat deposition and steatosis with accompanying hepatic and systemic insulin resistance. Therefore, prevention of diabetogenic tamoxifen side effects should include dietary advice or pharmacotherapy to prevent steatosis hepatitis.
